# Susceptibility to scrapie and disease phenotype in sheep: cross-*PRNP* genotype experimental transmissions with natural sources

**DOI:** 10.1186/1297-9716-43-55

**Published:** 2012-07-02

**Authors:** Lorenzo González, Martin Jeffrey, Mark P Dagleish, Wilfred Goldmann, Sílvia Sisó, Samantha L Eaton, Stuart Martin, Jeanie Finlayson, Paula Stewart, Philip Steele, Yvonne Pang, Scott Hamilton, Hugh W Reid, Francesca Chianini

**Affiliations:** 1 Animal Health and Veterinary Laboratories Agency, Pentlands Science Park, Bush Loan, Midlothian, EH26 0PZ, United Kingdom; 2 Moredun Research Institute, Pentlands Science Park, Bush Loan, Midlothian, EH26 0PZ, United Kingdom; 3 The Roslin Institute and R(D)SVS, University of Edinburgh, Easter Bush, Midlothian, EH25 9RG, United Kingdom; 4 Present address: UC Davis School of Veterinary Medicine, One Shields Ave, Davis, CA, 95616, USA

## Abstract

It has long been established that the sheep *Prnp* genotype influences the susceptibility to scrapie, and some studies suggest that it can also determine several aspects of the disease phenotype. Other studies, however, indicate that the source of infection may also play a role in such phenotype. To address this question an experiment was set up in which either of two different natural scrapie sources, AAS from AA_136_ Suffolk and VVC from VV_136_ Cheviot sheep, were inoculated into AA_136_, VA_136_ and VV_136_ sheep recipients (*n* = 52). The immunohistochemical (IHC) profile of disease-associated PrP (PrP^d^) accumulation in the brain of recipient sheep was highly consistent upon codon 136 homologous and semi-homologous transmission, but could be either similar to or different from those of the inoculum donors. In contrast, the IHC profiles were highly variable upon heterologous transmission (VVC to AA_136_ and AAS to VV_136_). Furthermore, sheep of the same *Prnp* genotype could exhibit different survival times and PrP^d^ profiles depending on the source of infection, and a correlation was observed between IHC and Western blot profiles. It was found that additional polymorphisms at codons 112 or 141 of AA_136_ recipients resulted in a delayed appearance of clinical disease or even in protection from infection. The results of this study strongly suggest that the scrapie phenotype in sheep results from a complex interaction between source, donor and recipient factors, and that the *Prnp* genotype of the recipient sheep does not explain the variability observed upon codon 136 heterologous transmissions, arguing for other genetic factors to be involved.

## Introduction

Classical scrapie is a transmissible spongiform encephalopathy (TSE) that occurs as a natural infectious and contagious disease of sheep and goats. It can also be transmitted experimentally by a variety of routes, not only to its natural host species but also to other mammals, notably laboratory rodents. The aetiological agent of scrapie is thought by many to be a prion, which is defined as an abnormal and infectious isoform of a cellular prion protein, PrP^c^[1], encoded by the host *Prnp* gene [2]. The abnormal isoforms accumulate during infection and may be defined operationally as protease-resistant PrP (PrP^res^) when detected by methods that use enzyme digestion, or disease-associated PrP (PrP^d^) when detected by methods such as immunohistochemistry (IHC).

It has long been established that susceptibility of sheep to scrapie is modulated by polymorphisms of the *Prnp* gene. While several polymorphisms may influence susceptibility, those at codons 136 and 171 -which in the wild-type allele encode alanine (A) and glutamine (Q), respectively- are understood to be particularly influential. Thus, sheep encoding valine (V) at codon 136 (VV_136_QQ_171_ and VA_136_QQ_171_) show enhanced susceptibility compared to AA_136_QQ_171_ animals, while those encoding arginine (R) at codon 171 (AA_136_RR_171_ and AA_136_RQ_171_) show increased resistance [3-6]. “In vitro” assays have led some researchers to postulate that such relative susceptibility or resistance may result from the efficiency by which different polymorphic variants of PrP^c^ can convert to PrP^res^[7].

Several studies on natural scrapie indicate that the sheep *Prnp* genotype, in particular at the two above mentioned codons, can influence not just the susceptibility to infection but also different aspects of the disease phenotype. These include survival times [8], clinical signs [9,10], vacuolar lesion profiles [11] and IHC patterns of PrP^d^ accumulation in the brain [12]. Other studies dealing with both natural and experimental sheep scrapie and with experimental bovine spongiform encephalopathy (BSE) in sheep have indicated that the TSE source, isolate, agent or strain may also play a role in the pathological [13] and IHC [14-19] phenotypes of the disease. Similarly, studies of transmission of TSE isolates to mice have shown that the murine disease phenotype (incubation period and vacuolar lesion profile) depends on an interaction between the TSE agent and host genetic factors [20,21]. Also, sporadic Creutzfeldt-Jakob disease of man shows different phenotypes that correlate with polymorphisms at codon 129 but, as is also found in sheep [16], two or more disease phenotypes can be recognised for a single *PRNP* genotype [22,23].

The experiment reported here aimed to determine the extent to and conditions in which the susceptibility to scrapie and the disease phenotype in sheep was influenced solely by *Prnp* genetic factors of the recipients or also by donor-related factors.

## Material and methods

### Experimental design

**Figure 1:**
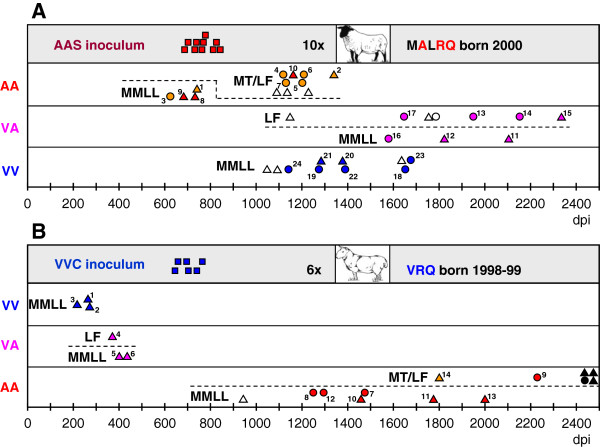
**Diagramatic representation of the global outcome of the experiment (excluding ARQ/ARR recipients and environmental controls).A**: transmissions with AAS inoculum. **B**: transmissions with VVC inoculum. Survival times in days (dpi) of AAS and VVC inoculum donors (red and blue squares, respectively), and of ARQ/ARQ (AA, red if Suffolk, orange if Cheviot), VRQ/ARQ (VA, pink, all Cheviot) and VRQ/VRQ (VV, blue, all Cheviot) recipients, challenged by the oral (triangles) or subcutaneous (circles) route. In white, PrP^d^-positive sheep dying from intercurrent conditions or culled for welfare reasons (all of them Cheviot in the case of AAS inoculum and Suffolk in the case of VVC inoculum). Black circle and triangles, four sheep (two Cheviot and two Suffolk) culled at the end of the experiment which resulted completely negative for PrP^d^ and PrP^res^ by IHC and WB, respectively. Dotted lines split sheep with additional polymorphisms at codons either 112 (MT, Suffolk) or 141 (LF, Cheviot) from MMLL sheep (with no additional polymorphisms). Numbers next to each symbol correspond to sheep identification numbers in Additional file 2.

Two different inocula were used to challenge a total of 65 sheep of two breeds and four different *Prnp* genotypes by two routes (Table 1). One inoculum (AAS) was prepared from the brains of 10 Suffolk sheep born in 2000 in a closed flock naturally affected by scrapie, the clinical, pathological and epidemiological aspects of which have been reported previously [14,16,24,25]. All those 10 sheep were *Prnp* AA_136_QQ_171_ genotype and developed clinical scrapie around two years after birth (Figure 1)A. The other inoculum (VVC) was prepared from the brains of six clinically affected Cheviot sheep born in 1998–1999 in another closed flock; the clinical, pathological and epidemiological aspects of scrapie in this flock have also been reported previously [26-28]. All those six sheep were *Prnp* VV_136_QQ_171_ genotype and developed clinical scrapie also at around two years after birth (Figure 1b). All donor and recipient sheep were homozygous for arginine (R) at codon 154. 

**Table 1:** Experimental design

**Recipient**	**AAS inoculum**		**VVC inoculum**		
**codon 136**	**po**	**sc**	**po**	**sc**	**Total**
Suffolk AA*	3sh		5 ht	5ht	13
Suffolk AA	3hm		5 ht	5ht	13
Cheviot AA	5 hm	5 hm	3 ht		13
Cheviot VA	5 sh	5 sh	3 sh		13
Cheviot VV	5 ht	5 ht	3 hm		13
Total	21	15	19	10	65

po, “per os” (oral route); sc, subcutaneous route. *codon 171 genotype of those recipients was RQ; all others were QQ. With regard to *Prnp* codon 136, transmissions are: hm, homologous; sh, semi-homologous; ht, heterologous.

Table 1 provides details of the inoculations with the two inocula in the different recipients by either the oral or subcutaneous route (5 g or 0.5 g tissue equivalent, respectively). The inoculations were carried out between September and November 2002, when the recipient sheep were 5–6 months old; these sheep were sourced from a New Zealand-derived, scrapie-free flock (ARSU, AHVLA, Addlestone, UK). In summary, the following inoculations were done: i) “Homologous transmissions”: VVC to three VV_136_QQ_171_ (VV) recipients and AAS to 13 AA_136_QQ_171_ (AA) recipients; ii) “Heterologous transmissions”: VVC to 13 AA recipients and to 10 AA_136_RQ_171_ (RQ) recipients, and AAS to 10 VV recipients; iii) “Semi-homologous transmissions”: VVC to three VA_136_QQ_171_ (VA) recipients, and AAS to 10 VA recipients and to 3 RQ recipients. A further 4 sheep (two AA Suffolk and two VV Cheviot) were kept as non-inoculated, environmental controls. In total, there were 17 sheep groups (15 inoculated –see Table 1- and two environmental controls) that were housed in as many different pens. Of the 65 inoculated sheep, 43 were castrated males and 22 were non-mated females.

It should be noted that, at the start of the experiment, the influence of polymorphisms at codons other than 136, 154 and 171 on the susceptibility to and pathogenesis of sheep scrapie was unknown; therefore, amino acids were determined only at those codons. In 2010, the full open reading frame of *Prnp* was determined retrospectively for most donor and recipient sheep in the experiment (see below).

### Monitoring of clinical disease and post-mortems

Monitoring for clinical signs of scrapie was carried out daily by a protocol described in detail in Additional file 1 (Table S1.1). Sheep reaching a defined clinical end point and those culled for welfare reasons were euthanised by barbiturate overdose and necropsies performed. The latter, as well as sheep that died from intercurrent disease, were included in the estimations of attack rates, but excluded from all other analyses performed. Sheep that did not develop clinical disease by the end of the experiment were culled at 2400–2500 days post-inoculation (dpi). All 65 sheep in the experiment and the environmental controls were subjected to the same necropsy protocol and range of laboratory examinations.

### Tissue sampling and immunohistochemistry (IHC)

At post-mortem, samples of the central nervous system (CNS) were collected, including the whole brain and three levels of the spinal cord. The brain was halved and one hemi-brain was frozen at −80°C for biochemical studies and the other fixed in formaldehyde for IHC examinations. Following fixation, 7 different brain areas (frontal cerebral cortex, corpus striatum/basal ganglia, thalamus/hypothalamus, midbrain, cerebellar cortex/peduncles, rostral medulla oblongata, and medulla at the obex) and 3 levels of the spinal cord (3^rd^ cervical, 10^th^ thoracic and 3^rd^ lumbar) were processed for IHC examination of PrP^d^ with the rat monoclonal antibody R145, which binds to amino acid sequence RESQA (222–226) of ovine PrP (AHVLA, Addlestone, UK), by methods previously described [14,15].

PrP^d^ scoring in CNS samples, of both inocula donors and recipient sheep, was carried out using the PrP^d^ profiling method previously described [14,15]. Briefly, the method involved the scoring from 0 to 3 of five different patterns of PrP^d^ accumulation: i) intraneuronal, ii) intraglial (intramicroglial and intrastrocytic types combined), iii) extracellular glia-associated (subpial, subependymal, perivascular, stellate and perivacuolar types combined), iv) grey matter neuropil-associated (diffuse particulate, coalescing, perineuronal and linear types combined), and v) other types (ependymal, vascular plaque and non-vascular plaque types combined) at the 7 brain areas and 3 spinal cord levels mentioned above.

### Western blotting (WB)

Samples of caudal medulla from all 65 recipient sheep were examined for the detection of PrP^res^ by WB with P4 and SAF84 monoclonal antibodies (R-biopharm, Darmstadt, Germany), which bind to amino acid sequences 93–99 and 160–170 of ovine PrP, respectively. These samples were analysed once and each gel included the same positive and negative controls to ensure consistency of results between different gels.

Samples were treated as described previously [29], with some minor refinements. Briefly, samples were homogenised at 20% (w/v) in lysis buffer and frozen at −20°C overnight. Lysates were then diluted to 10% in lysis buffer and then digested with 50 μg/mL proteinase K solution for 1 h, at 37°C in agitation. Digestion was terminated by adding 1 mM Pefabloc SC (Roche Diagnostics, Burgess Hill, West Sussex, UK). Samples were then centrifuged at 20 000 × *g* for 1 h at 4°C, the supernatants discarded and the pellets resuspended in 45 μL 2 × Sample Buffer (Invitrogen, Paisley, UK) containing 5 μL of 10 × sample reducing agent (Invitrogen, Paisley, UK). Samples were heated at 100°C for 5 min and once cooled pulsed for 5 s at 5000 rpm. SDS-PAGE was carried out on 10 μL of sample on 4-12% Bis-Tris NuPAGE gels (Invitrogen, Paisley, UK) at 200 V for 40 min. Proteins were electrotransferred onto Hybond P PVDF membrane (GE Healthcare, Chalfont St Giles, Buckinghamshire, UK) at 30 V for 1 h. Non-specific antigen binding on the membrane was blocked by soaking in 2% non-fat milk/TBS with 0.1% Tween_20_ (Sigma Chemical Company, Poole, Dorset, UK) and the membranes were probed with either P4 or SAF 84 antibodies. Signal detection and analysis were performed as previously reported [29].

### *Prnp* genotyping

At the start of the experiment, amino acids at codons 136, 154 and 171 of the ovine PrP were determined by sequencing with an ABI Prism 377 DNA sequencer according to the manufacturer’s instructions (PE Applied Biosystems; Warrington, UK). In 2010, and in order to ascertain the existence of polymorphisms at other codons, blood or brain tissue samples from 52 recipient sheep (all except the 13 RQ), the 10 donors of the AAS inoculum and the 4 environmental controls were taken for PCR amplification and sequencing of the whole open reading frame of the *Prnp* gene on an 3130 Genetic Analyzer with the BigDye® terminator v3.1 cycle sequencing kit as per the manufacturer’s protocol (PE Applied Biosystems).

### Statistical analyses

Differences between transmission groups (defined as the combination of inoculum source and *Prnp* genotype of recipient) in terms of survival times were analysed by non-parametric unpaired *t* tests (Mann–Whitney; Instat® GraphPad Softaware, San Diego, USA). The same analyses were used to determine differences in WB features (molecular weights of the unglycosylated band and glycoprofiles) between sheep with different PrP^d^ profiles determined by IHC examinations. Differences in attack rates and frequency of presentation of different clinical signs between transmission groups were analysed by Fisher’s exact test (Instat® GraphPad Softaware).

## Results

### Attack rates, survival times and clinical signs

None of the 13 RQ sheep, either dosed with AAS or VVC inocula, developed any clinical signs suggestive of scrapie on routine examinations and were consistently negative in three serial rectal biopsy examinations performed over a period of 18 months (results not shown). Two of them died from intercurrent conditions at 1200 and 1500 dpi and the remaining 11 were culled at 2200 dpi. None of them showed any evidence of PrP^d^ or PrP^res^ accumulation in any of the tissues examined by IHC and WB, respectively. Similarly, the 4 environmental controls were fully negative when culled at the end of the experiment. None of those 17 sheep showed any additional polymorphism at any *Prnp* codon, and neither did any of the sheep providing the AAS and VVC inocula.

Of the remaining 52 recipients (Figure 1), none of the 13 VV Cheviot recipients showed additional polymorphisms. In contrast, 8 of the 13 VA and 11 of 13 AA Cheviot sheep recipients were LF_141_ (L, leucine, F, phenylalanine), and 4 of the 13 AA Suffolk sheep were MT_112_ (M, methionine, T, threonine).

A total of 48 of those 52 recipients were diagnosed as scrapie-infected at post-mortem. Attack rates reached 100% in all donor/recipient combinations, regardless of the route of infection, with only one exception: the heterologous VVC to AA transmissions, for which the attack rate was 69.2% (9/13). The four PrP^d^ and PrP^res^ negative sheep (one inoculated sc) were long-term survivors culled at the end of the experiment, more than 2400 dpi. This transmission group could be split into two: seven recipients with no additional polymorphisms, all of which were scrapie positive at post-mortem (100% attack rate), and six animals with additional polymorphisms (three MT_112_ and three LF_141_); of these, two each were negative and one each positive (33% attack rate). The two attack rate figures of non-polymorphic and polymorphic recipients were significantly different in the Fisher’s exact test (*P* = 0.02).

Of the 48 scrapie positive sheep, 10 died from intercurrent conditions, either after having shown early clinical signs of scrapie, or in their complete absence; these sheep did not belong to any particular transmission group (Figure 1). Amongst the remaining 38 sheep, all of which reached clinical end point, survival times were shortest in VVC homologous and semi-homologous transmissions followed by AAS homologous transmission to AA sheep without additional polymorphisms. The longest survival times were observed in semi-homologous AAS to VA transmissions and in the two polymorphic AA recipients challenged with VVC inoculum. Table 2 provides details of survival times and of the statistical differences between transmission groups (individual details can be found in Additional file 2). A number of non-ideal assumptions were made to obtain groups large enough to allow statistical analysis; in our opinion such compromises are justified in view of the actual data, as follows:

**Table 2:** Statistical analysis of differences in survival times

	**Survival Times**	**AAS to**				**VVC to**			
		**AA(4)**	**AA+(6)**	**VA(7)**	**VV(7)**	**VV(3)**	**VA(3)**	**AA(6)**	**AA+(2)**
		**679 ±54**	**1195±90**	**1934±275**	**1400±200**	**245±33**	**399±28**	**1544±294**	**2023±303**
AAS to									
AA(4)	679±54								
AA+(6)	1195±90	**							
VA(7)	1934±275	**	**						
VV(7)	1400±200	**	*	**					
VVC to									
VV(3)	245±33	n/a	*	*	*				
VA(3)	399±28	n/a	*	*	*	n/a			
AA(6)	1544±294	**	**	*	ns	*	*		
AA+(2)	2023±303	n/a	n/a	n/a	n/a	n/a	n/a	n/a	

AA+, sheep with additional polymorphisms at codons 112 or 141. Survival times are indicated as mean ± SD. In brackets, numer of clinically affected sheep within each transmission group. n/a, analysis not performed due to insufficient sheep numbers. Ns, no significant differences; * *P* < 0.05; ** *P* < 0.01 in the Mann–Whitney test.

- The two non-polymorphic AA Cheviot sheep inoculated with AAS were grouped together with the two non-polymorphic AA Suffolk sheep, as their survival times were very similar (Figure 1). For the same reason the only polymorphic AA Suffolk recipient was considered together with the five polymorphic Cheviot sheep, all challenged with AAS. Similarly, the only clinically affected, polymorphic AA Cheviot and AA Suffolk sheep inoculated with VVC were grouped together in view of their protracted incubation periods; this arrangement had no effect on the statistical analyses, as the group was still too small (Table 2).

- VA recipients of each of the two semi-homologous transmission groups were considered as a single group regardless of the presence or absence of additional polymorphisms, since these polymorphisms appeared not to have any effect on survival times in any of the two transmission groups (Figure 1). In fact, the only polymorphic VA sheep receiving VVC inoculum had the shortest, though similar, survival time of this group.

- AA recipients either with MT or LF polymorphisms were considered together as AA sheep with additional polymorphisms (AA + in Table 2). Both polymorphisms had the same effect on the attack rate after VVC challenge (33%), and the survival time of the MT Suffolk sheep in the homologous transmission group was practically the same as that of four of the five LF Cheviot sheep (Figure 1).

- In some transmission groups, sheep inoculated subcutaneously appeared to have slightly shorter survival times than those dosed orally (eg. AAS to VA or VVC to non-polymorphic AA, the survival times of which were very long and spread regardless of route of challenge), while in other groups the opposite effect (AAS to VV) or no effect (AAS to AA) was found (Figure 1). In view of this the two routes were grouped together.

- Males and females showed no differences in attack rates or survival times (data not shown).

With these assumptions in mind, the two key findings of the analysis of survival times were (Table 2):

- The influence of the source of inoculum for each of the different recipient genotypes. Thus, AA recipients without additional polymorphisms showed significantly shorter survival times when challenged with AAS (679 ± 54 [meandays ± SD]) than in transmissions with VVC inoculum (1544 ± 294; *P* < 0.01), while VV and VA sheep challenged with VVC had significantly shorter survival times (245 ± 33 and 399 ± 28, respectively) than in transmissions with AAS inoculum (1400 ± 200 and 1934 ± 275, respectively; *P* < 0.05 in both cases).

- For AA recipients challenged with homologous AAS inoculum, additional polymorphisms, which resulted in significantly longer survival times (1195+/-90) compared to non-polymorphic sheep (679+/-54; P<0.01). The influence of such polymorphisms could not be tested in VVC to AA transmissions, as only two of these recipients were polymorphic; their survival times were however very protracted (1809 and 2237 days).

Details of the frequency and severity of the different types of scrapie-associated clinical signs are given in Additional file 1 (Table S1.2). Ataxia was the predominant clinical sign in all transmission groups, as it was shown by 89% of the affected sheep, while weight loss was the least common of the clinical signs (24% of cases). Signs of pruritus were recorded in 67% of cases in which the VV genotype was present, either as inoculum or as recipient, which was in contrast with 24% of cases in which those signs were observed in the absence of the VV genotype. Conversely, dysphagia correlated negatively with the VV genotype (see details in Additional file 1, Table S1.2).

### Phenotype of PrP^d^ accumulation in the CNS

**Figure 2:**
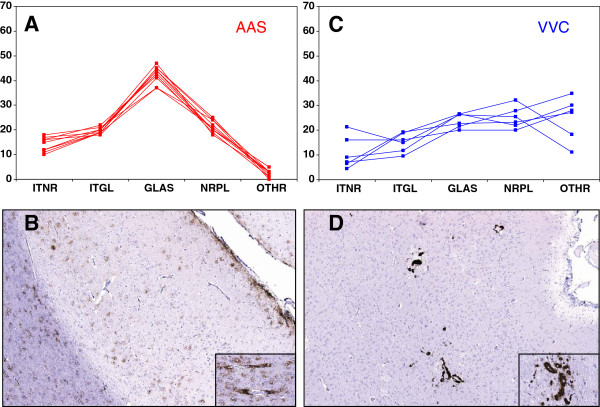
**Profiles of PrP**^**d**^**accumulation in the brain of inocula donors.A**, **B**: AAS: note the high degree of similarity between the individual profiles (**A**), which are characterized by prominent extracellular deposits, particularly those associated to glial cells (A type; sub-pial, stellate, peri-vascular and perivacuolar, as illustrated in **B**; inset, detail of peri-vascular and peri-vacuolar aggregates in white matter of the cerebral cortex). **C**, **D**: VVC: note slight more variable and clearly different profiles (**C**) than in AAS donors, with prominence of vascular plaques in cerebral cortex (X type, as illustrated in **D**; inset, detail of intramural and perivascular plaques in corpus striatum). IHC with R145 PrP monoclonal antibody and haematoxylin counterstaining. Original magnifications: large images × 4, insets × 20). PrP^d^ types (X-axis) are: ITNR: intraneuronal; ITGL: intraglial; GLAS: glia-associated extracellular; NRPL: extracellular in grey matter neuropil; OTHR: other types. For more detailed description see text. Y-axis indicates proportion of the different PrP^d^ types.

The PrP^d^ profiles in the brains of donors are summarised and illustrated in Figure 2. All the 10 Suffolk sheep that provided the AAS inoculum showed very similar profiles of PrP^d^ accumulation in the brain (profile type A; Figure 2a). This was characterised by relatively low amounts of intracellular PrP^d^ (intraneuronal and intraglial), prominent deposits of extracellular protein in association with glial cells (in both grey and white matter; Figure 2b), and to a lesser extent in the grey matter neuropil; other types of PrP^d^ found were in most instances negligible and restricted to deposits in ependymal cells. A higher degree of variability was observed amongst the six donors of the VVC inoculum (Figure 2c), which showed variable amounts of intracellular PrP^d^, moderate amounts of extracellular protein and significant levels of vascular plaques (profile type X, Figure 2d).

**Figure 3:**
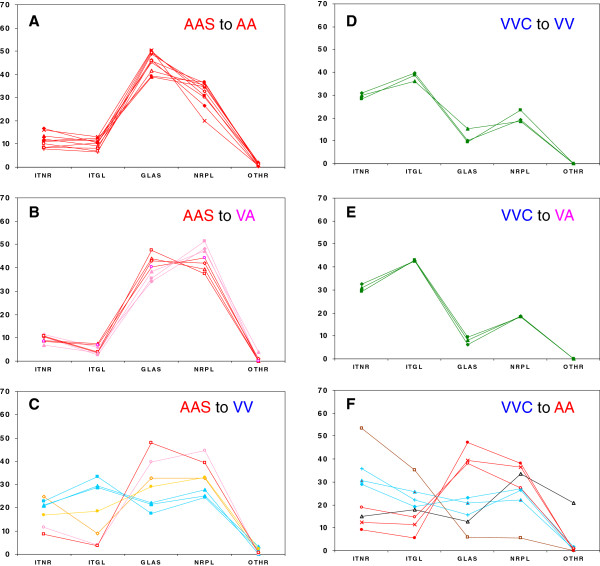
**Profiles of PrP**^**d**^**accumulation in the brain of recipients of either AAS (A-C) or VVC (D-F) inoculum.** Individual profiles that show high degree of similarity are identified by the same colour (red, type A; pink, type A’; green, type M; light blue, type M’; gold, type U; orange, type U’; black, type P; brown, type CH). Note the high degree of variability amongst recipients of codon 136 heterologous transmissions (**C** and **F**), which contrasts with the consistency of profiles amongst homologous and semi-homologous transmissions recipients for both AAS (**A** and **B**) and VVC (**D** and **E**) inocula. PrP^d^ types (X-axis) are: ITNR: intraneuronal; ITGL: intraglial; GLAS: glia-associated extracellular; NRPL: extracellular in grey matter neuropil; OTHR: other types. For more detailed description see text. Y-axis indicates proportion of the different PrP^d^ types.

**Figure 4:**
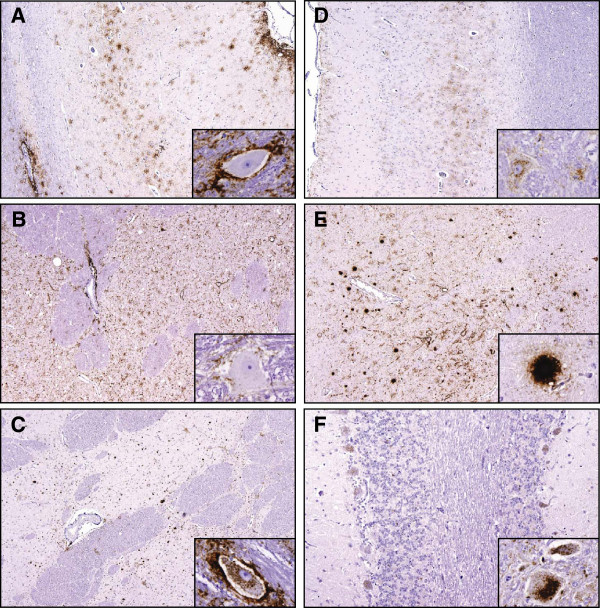
**Examples of the brain PrP**^**d**^**profile types encountered in different transmission groups.A**: recipient AAS1: type A profile in cerebral cortex (note similarities with Figure 2a); inset, recipient AAS10 (type A) illustrating the absence of intracellular PrP^d^ in deep cerebellar nuclei despite the presence of abundant extracellular aggregates. **B**: recipient AAS17: type A’ profile in corpus striatum: peri-vascular PrP^d^ in capsula interna and abundant extracellular (peri-neuronal, linear and particulate) PrP^d^ in neuropil but inconspicuous intraneuronal PrP^d^ in deep cerebellar nuclei (inset, same recipient). **C**: recipient VVC11 (type M’) showing co-occurrence of extracellular (linear, perineurinal, particulate and coalescing) and intraneuronal PrP^d^ in corpus striatum; inset: recipient VVC12 showing the same co-occurrence of intra- and extra-cellular PrP^d^ in deep cerebellar nuclei (type M'). **D**: recipient VVC1: type M profile with almost complete absence of extracellular glia-associated PrP^d^ and some neuropil-associated and intracellular aggregates in deep cerebral cortex layers; inset, same recipient showing abundant intraneuronal PrP^d^ in deep cerebellar nuclei. **E**: recipient VVC13 (type P profile) showing abundant extracellular PrP^d^ in the neuropil of the thalamus and prominent non-vascular, Kuru-type plaques (detail in inset). **F**: recipient VVC14 (type CH profile) showing conspicuous intracellular PrP^d^ deposits in Purkinje cells and in neurons and glial cells within the red nucleus (inset). IHC with R145 PrP monoclonal antibody and haematoxylin counterstaining. Original magnifications: large images × 4 (except F, ×10); insets × 60.

The PrP^d^ profiles in the brains of the recipients are summarised in Figure 3 and illustrated in Figure 4 (detailed values of the different PrP^d^ types are provided in Additional file 2). In the transmissions with the AAS inoculum, the PrP^d^ profile was homogeneous amongst all AA recipients (profile type A, sheep AAS1-10, Figures 3a and 4a) and closely resembled that of the donors (Figures 2a and 2b). This consistency was independent of differences in breed (3 Suffolk and 7 Cheviot), route of infection (5 oral and 5 subcutaneous) and additional polymorphisms (4 non-polymorphic and 6 polymorphic). A consistent PrP^d^ profile was found for all VA recipients (Figure 3b) which closely resembled that of the donors (profile type A, sheep AAS11-14), with the exception of three of them in which extracellular PrP^d^ in the neuropil was slightly more prominent than extracellular glia-associated PrP^d^ (profile type A’, sheep AAS15-17, Figure 4b). A high degree of variability was observed in the heterologous transmission to VV recipients (Figure 3c): one sheep showed type A profile (AAS18), although with a few vascular plaques, another sheep showed a type A’ profile (AAS19), three were clearly different, showing similar levels of intra- and extracellular PrP^d^ (profile type M’, sheep AAS20-22, Figure 4c) and two showed intermediate profiles (profile types U and U’, sheep AAS23 and 24, respectively, not illustrated), including one sheep in which a few non-vascular plaques were present. This variability could not be attributed to differences in route of infection (see individual details in Additional file 2).

In the transmissions with the VVC inoculum, the PrP^d^ profile was very similar amongst all VV (VVC1-3) and VA (VVC4-6) recipients (homologous and semi-homologous transmission, Figure 3d and 3e, respectively), with predominance of intraneuronal and, particularly, intraglial deposits and very low levels of extracellular glia-associated PrP^d^ (profile type M, Figure 4d). However, they did not resemble the profiles of the inoculum donors (Figure 2c and 2d), as they showed clearly higher levels of intracellular PrP^d^ and absence of vascular plaques. Again, a high degree of variability was observed in the heterologous transmission to AA recipients (Figure 3f): three were reminiscent of the AA recipients challenged with AAS inoculum (type A, VVC7-9, the first showing a few vascular plaques in cerebral cortex), and three had similar levels of extra- and intracellular deposits (VVC10-12), thus resembling some of the VV recipients dosed with AAS inoculum (type M’). The profiles of the last two sheep of this transmission group did not resemble those of other donor or recipient sheep: one showed abundant extracellular deposits of PrP^d^ in the grey matter neuropil and distinctive non-vascular plaques throughout the brain (type P, VVC13, Figure 4e), and the other displayed very prominent intraneuronal and intraglial aggregates being therefore reminiscent of experimental CH1641 scrapie (VVC14, profile type CH, Figure 4f). The variability in PrP^d^ profiles of AA recipients did not appear to result from differences in route of inoculation or, in the case of Suffolk sheep, additional polymorphisms (see details in Additional file 2).

An analysis carried out to assess the relationship between the IHC phenotype in the brain and the clinical signs exhibited by the animals (see Additional file 2 for individual details) indicated that pruritus had been recorded in 7/23 (30%) sheep with IHC types A or A’, a proportion significantly lower (*P* < 0.05; Fisher’s exact test) than in those with M or M’ PrP^d^ profiles (9/12, 67%). In contrast, signs of dysphagia were observed in a higher proportion of sheep with A or A’ brain profiles (14/23, 61%) than of those with M or M’ IHC types (0/12; *P* < 0.001).

### Western blotting in samples of obex

**Figure 5:**
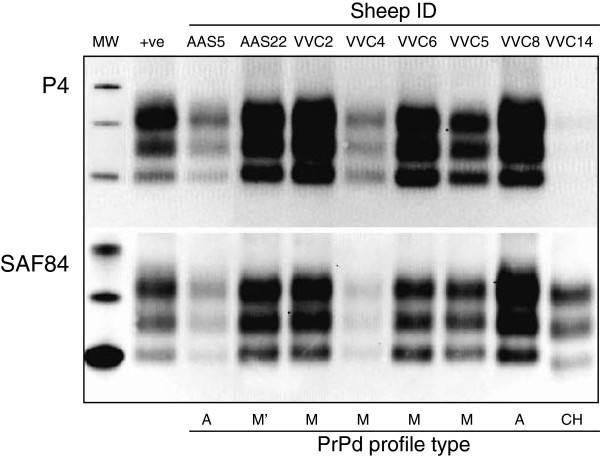
**Illustrative Western blot with P4 and SAF84 antibodies in samples of obex.** For individual identification of the eight sheep refer to Additional file 2. The IHC profile types for each brain are provided at the bottom of the illustration. VVC14 is the sheep with a CH1641-like PrP^d^ profile.

Positive PrP^res^ signal was detected by WB in 45 of the 48 sheep that were positive for PrP^d^ by IHC in the obex. The three WB negative sheep were amongst the 10 intercurrent deaths/welfare culls, and two of them showed just trace accumulations of PrP^d^ in the brain. All the 38 clinically affected sheep provided positive WB reaction with both P4 and SAF84 antibodies with the exception of one animal, the obex sample of which did not react with P4 and showed a low molecular weight (18.7 KDa) unglycosylated band with SAF84 (Figure 5); this sheep (VVC14) was the AA Cheviot orally dosed with VVC inoculum that provided a CH1641-like PrP^d^ profile in brain.

**Figure 6:**
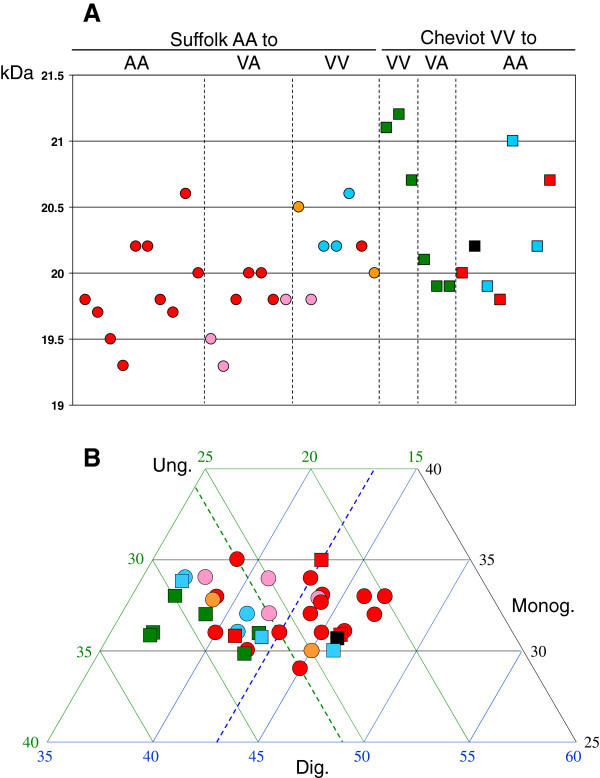
**Molecular characteristics of PrP**^**res**^**(WB with P4 antibody) in obex samples.** Sheep recipients of the different transmission groups are colour-coded to match the IHC profiles shown in Figure 3. **A**: Molecular weights (MWs in kDa) of the unglycosylated band: note the association between AAS inoculum (circles), A and A’ PrP^d^ profiles (red and pink, respectively) and generally lower MWs, and between VVC inoculum (squares), M and M’ IHC profiles (green and light blue, respectively) and generally higher MWs (for statistical analysis refer to text). **B**: Triplot representation of the glycoprofiles as proportion of unglycosylated (ung., green scale and grid), monoglycosylated (monog, black scale and grid) and diglycosylated band (dig, blue scale and grid): note that 11 of 12 sheep with M or M’ IHC profiles have more than 26% of unglycosylated PrP^res^, whereas 18 of 23 with A or A’ PrP^d^ profiles have 26% or less unglycosylated PrP^res^ (dotted green line). Similarly, 11 of 12 sheep with M or M’ IHC profiles have less than 43% of diglycosylated PrP^res^, whereas 15 of 23 with A or A’ PrPd profiles have 43% or more diglycosylated PrP^res^ (dotted blue line). For statistical analyses refer to text. The only one sheep with a CH type of PrP^d^ profile (see Figure 3) is not represented as it did not react with P4 antibody in the WB.

In the analysis of the different inoculum/recipient combinations, differences were observed both in respect of molecular weights (MWs) of the unglycosylated bands and of glycoprofile. These differences, however, were only evident with P4 antibody (see values in Additional file 2) and not with SAF84 (data not shown). As indicated earlier, the interaction between the *Prnp* genotype of the recipient and the source of inoculum resulted in a variety of PrP^d^ brain profiles, which were shown to be associated with molecular profiles. Thus, while no differences in MWs were found between A and A’ or between M and M’ IHC types, the latter showed significantly higher MW unglycosylated bands (M + M’, 20.4 ± 0.1) than the former (A + A’, 19.9 ± 0.1; *P* = 0.001; Figure 6a). In terms of glycoprofile, differences were only found in the proportion of di- and unglycosylated bands (Figure 6b), so that sheep with A or A’ PrP^d^ profile types showed significantly higher diglycosylated PrP^res^ than sheep with M or M’ IHC profiles (43.2 ± 0.6 vs 39.8 ± 0.8; *P* < 0.01) and significantly lower levels of unglycosylated PrP^res^ (24.3 ± 0.5 vs 28.2 ± 0.6; *P* < 0.001).

## Discussion

When measuring the efficiency of transmission in terms of attack rate and survival time, the results of this experiment showed that the degree of genotypic homology between donors and recipients is a key factor in the development of scrapie. Thus, homologous transmission both with VVC inoculum to VV recipients and with AAS inoculum to non-polymorphic AA recipients resulted in markedly shorter survival times than their heterologous counterparts. The fact that VV and VA recipients infected with AAS showed more protracted incubation periods than AA sheep challenged with the same inoculum contradicts the widespread notion that sheep of the VRQ/VRQ and VRQ/ARQ *Prnp* genotypes are the most susceptible to scrapie [30]. Moreover, additional polymorphisms at codons either 112 or 141 in homozygous AA Suffolk or Cheviot sheep, respectively, resulted in extended survival times in transmission with AAS inoculum and an incomplete attack rate in heterologous transmission with VVC inoculum. The effect of the T_112_ polymorphism has been previously documented for natural [31] and experimental scrapie [32], and also for experimental BSE [33], and may be related to the low PrP^c^ to PrP^res^ conversion efficiency of this protein variant observed “in vitro” [34]. In contrast, a similar effect of the F_141_ polymorphism has only been described in sheep orally dosed with BSE [35] but not convincingly for classical scrapie. Based on a non-significant reduction effect of the F_141_ polymorphism on the “in vitro” convertibility of PrP^c^ to PrP^res^, a neutral effect of such polymorphism on the susceptibility to scrapie was proposed [34]. Our study clearly shows that, for AA recipients, even when the F_141_ polymorphism is in heterozygosity it affects susceptibility to classical scrapie. However, none of those additional polymorphisms appeared to have a similar effect in VA heterozygotes, whose behaviour was entirely dependant of the source of inoculum. While those challenged with VVC inoculum had survival times just slightly longer than VV recipients and clearly shorter than AA recipients, those challenged with AAS inoculum showed the longest survival times, even longer than VV recipients inoculated with the same AAS source (heterologous transmission). This paradoxical situation (sometimes referred to as over-dominance) may be the response to a strain- or source-dependant allelic interference phenomenon, which has been previously documented in some murine TSE models [36] and in scrapie-infected VRQ/AHQ heterozygotes [37]. The same phenomenon could perhaps explain the protracted incubation period of VA_136_RQ_171_ compared to VV_136_QQ_171_ sheep after SSBP/1 infection [38] or in natural conditions [39].

Non-polymorphic AA and VV recipients showed different behaviour in their respective homologous challenges. Thus, AA recipients succumbed to scrapie with incubation periods (~700 days) and brain IHC phenotypes (type A) similar to those observed in the naturally affected sheep that provided the AAS inoculum (see Figures 1a2a and 3a). In contrast, the VV recipients developed scrapie with a much shorter incubation period (~250 days) than that observed in the natural cases providing the VVC inoculum (~700 days, assuming infection at around birth; see Figure 1)b, and also their brain PrP^d^ profile was, although consistent (type M; see Figure 3d), clearly different from that of the donors (type X; see Figure 2c). It has been proposed that the higher efficiency of the oral route compared to the natural infection, both in terms of PrP^d^ accumulation dynamics in brain and incubation period, is possibly due to differences in dose and exposure to infection between these two scenarios [40]. The results of the homologous transmission to AA sheep of our study does not support a consistent or necessarily higher efficiency of the oral route compared to natural infection but could, at least partially, explain the differences between VV donors and recipients. A different explanation is however possible, particularly in view of the differences in brain PrP^d^ IHC phenotype: that the VV inoculum contained a mixture of strains and that one prevails in low dose natural infection while a different one is responsible for disease progression after high dose oral infection. This hypothesis would be in agreement with the previously reported isolation of two different murine scrapie strains, ME7 and 221 C, from VRQ/VRQ sheep of the farm providing the VVC inoculum of this experiment [41].

With regard to the clinical signs observed in this experiment, the positive and negative associations of the VV genotype with signs of pruritus and dysphagia, respectively, are in agreement with those found in natural scrapie cases [10]. From the brain PrP^d^ profiles obtained in this experiment it would further appear that predominantly extracellular deposits (types A and A’) would be associated with signs of dysphagia, while more prominent intracellular PrP^d^ aggregates (types M and M’) would be associated with pruritus. However, the interpretation of this apparent association has to be cautious as, for example, the sheep with a CH1641-like profile (almost 90% of intracellular PrP^d^) did not show evident pruritus.

It has been suggested that, for natural scrapie, the IHC PrP^d^ profiles in the brain are mainly driven by the sheep *Prnp* genotype [11,12], and that the same factor is key in determining the lesion profile in mice inoculated with natural scrapie isolates [42,43]. In contrast, other studies have shown that variability of brain PrP^d^ profiles can occur within naturally infected sheep of a single genotype [16], confirming previous experimental results that indicated that the source of infection can also play a role in the pathological phenotype [14,15,17-19]. The results of the present experiment clearly support the latter notion since: i) sheep recipients of the same genotype can have different profiles, depending on the infecting source (compare, for example, VA sheep infected with the two inocula in Figure 3), ii) on cross-genotype, heterologous transmission, sheep recipients of the same genotype, either VV or AA, can show a diversity of profiles, and iii) sheep of different genotypes can show similar profiles not only when infected with the same source, but also when challenged with different sources. In summary, the IHC examination of the brain of clinically affected sheep within this study suggests that homologous and semi-homologous genotype transmission of scrapie results in consistent brain PrP^d^ profiles in the recipients. These can be indistinguishable from (AAS to AA and VA) or different (VVC to VV and VA) the profile of the source, possibly depending on the presence of one or more strains of the agent in the inoculum, as already mentioned. In contrast, codon 136 heterologous transmissions result in a diversity of brain PrP^d^ profiles, which in some cases appear to be driven by the source of inoculum, in others by the host genotype and in others again by neither of these factors. The emergence of P-type and H-type profiles (VVC13 and VVC14, respectively) in AA recipients challenged with VVC is an example of the latter. The finding of a CH1641-like profile (CH-type) is not surprising since the isolation of this experimental source originated from a VRQ/VRQ sheep from the same flock as the VVC inoculum donors used in this experiment [19]. It is worth noting that this IHC phenotype appeared in the only LF_141_ sheep of this transmission group, although any extrapolation on the basis of a single example would be premature.

Correlations were found between IHC and molecular profiles. Thus, predominantly extracellular PrP^d^ (types A and A’) is associated with more abundant diglycosylated PrP^res^ and little unglycosylated protein of slightly lower molecular weight, while prominent intracellular PrP^d^ (types M and M’) correlates with more abundant unglycosylated WB bands of higher molecular weight. One possible explanation would be that, while extracellular PrP^d^ is mostly intact, full length [18] and fully glycosylated, once it is internalised in the endosomal/lysosomal system it is not only cleaved at the N-terminus but also sugar residues are digested. Another explanation would be that in some source/host combinations, conversion of PrP^c^ to PrP^d^ takes place, at least partially, inside the cell, before glycosylation of PrP^c^ takes place; this would be in agreement with reports demonstrating that unglycosylated PrP^c^ is mainly intracellular and fully functional [44].

In conclusion, the disease phenotype arising in the natural sheep host upon experimental transmission appears to result from a complex interaction between donor and recipient factors. Amongst the donor factors, the *Prnp* genotype and the nature of the strain (and its presence as single or multiple strains) in the inoculum are likely to play a role; amongst the recipient factors the *Prnp* genotype does not explain all of the variability observed, arguing for other, probably genetic, factors to be involved. Whether or not the consistency or variability of disease phenotypes found in sheep bears significance in terms of strain diversity will be better understood by studying their correlation with other biological properties, for which bioassays in rodent models are currently underway.

## Competing interests

The authors declare that they have no competing interests.

## Authors’ contributions

LG participated in the design and coordination of the experiment, performed post-mortem and immunohistochemical (IHC) examinations, analyzed the data and drafted the manuscript. MJ conceived the study, participated in its coordination and helped to draft the manuscript. MPD carried out post-mortem examinations and tissue sampling. WG performed ORF *Prnp* genotyping. SS participated in post-mortems, tissue processing and IHC examinations. SLE helped with tissue sampling at post-mortem and carried out Western blot (WB) analyses. SM coordinated IHC processing and quality control. JF helped with clinical monitoring and tissue sampling at post-mortem. PaS helped with ORF *Prnp* genotyping. PhS helped with WB analyses. YP co-ordinated tissue sample collection at post-mortem and helped with WB analyses. SH helped with tissue sampling at post-mortem and WB analyses. HWR participated in the coordination the study and performed clinical monitoring. FC participated in the coordination the study, post-mortem examinations and data analysis. All authors read and commented on manuscript drafts and approved its final version.

## Supplementary Material

Additional file 1 **Clinical monitoring and clinical signs.** Details of clinical examinations performed and of frequency and severity of different scrapie-associated clinical signs. Click here for file

Additional file 2 **Individual details of 38 recipients that reached clinical end point grouped by their transmission group.** Individual sheep data on source of inoculum, route of inoculation, Prnp genotype, breed, survival time, clinical signs, IHC profiles and WB results Click here for file
