# Supplementary material for: Susceptibility to scrapie and disease phenotype in sheep: cross-PRNP genotype experimental transmissions with natural sources
Source: Vet Res. 2012 Jul 2;43(1):55. doi: 10.1186/1297-9716-43-55 (PMC3460791; doi:10.1186/1297-9716-43-55)
Supplement: Additional file 1 — Clinical monitoring and clinical signs. Details of clinical examinations performed and of frequency and severity of different scrapie-associated clinical signs. [file 1297-9716-43-55-S1.doc]

**Supplementary File 1 Clinical monitoring and clinical signs**

**Clinical monitoring**

Monitoring for clinical signs of scrapie was carried out daily. Visual inspection was performed and response to different stimuli and body condition assessed in order to detect signs of i) locomotor dysfunction/ataxia, ii) behavioural changes (from dullness to excitability), iii) dysphagia (ptyalism to cud dropping), iv) pruritus (rubbing, wool loss, scratch test), and v) weight loss (body condition score from 1 to 5). These clinical signs were scored from 0 to 3 (see details in Table S1.1), and clinical end point was established when any of those five groups of signs was given a score of 3 or when a combined score of 8 was reached.

**Clinical signs**

Details of the frequency and severity of the different types of scrapie-associated clinical signs are given in Table S1.2 Ataxia was the predominant clinical sign in all transmission groups, as it was shown by 89% of the affected sheep (88% for sheep challenged with AAS inoculum to 93% for those with VVC inoculum, and 89%-90% for recipients of the three different genotypes). Weight loss was the least common of the clinical signs (24% of cases; from 0% to 40% for VV and VA recipients, respectively, and no variation between inocula). Signs of dysphagia were shown by 37% of affected sheep (range from 0% to 56% for VV and AA recipients, respectively), pruritus by an average 47% of sheep (range from 33% to 80% for AA and VV recipients, respectively), and behavioural changes by 58% of animals (range from 43% to 67% for VVC and AAS inocula, respectively). The severity of the last three clinical signs (dysphagia, pruritus and behavioural changes) was mild when present (between 1.2 and 1.6 out of a maximum score of 3), whereas ataxia was the most severe clinical sign (average score of 2.8).

An association was observed between the appearance of signs of pruritus and the VV genotype, either from the source inoculum or from the recipient. Thus, while 14/21 (67%) recipients, either of any genotype and challenged with VVC or of VV genotype and challenged with any of the two sources, showed signs of pruritus, only 4/17 (24%) AA or VA recipients challenged with AAS did so (*P* = 0.01 in the Fisher’s exact test). In contrast, the VV genotype showed a negative correlation with the development of signs of dysphagia. When comparing the same groups as above, dysphagia was present in 2/21 (10%) of cases in which the VV genotype was involved either as source or recipient, while it was noted in 12/17 (71%) of cases in which such genotype was not involved (*P* < 0.01 in the Fisher’s exact test).

**Table S1.1. Scoring system for scrapie-associated clinical signs.**

Clinical sign Score

1 2 3

Ataxia wide-based stance abnormal gait recumbency

Behaviour mild dullness/excitability profound dullness/excitability unresponsiveness./seizures

Dysphagia wet mouth ptyalism/cud dropping unable to swallow

Pruritus rubbing rubbing and wool loss skin excoriations

Weight loss* 1 point loss in 2 months 1 point loss in 1 month 2 point loss in 1 month

or a body condition score of 1

*1 point ~10% body weight loss.

**Table S1.2. Frequency of presentation and severity of clinical signs associated with scrapie.**

Inoculum Recipient Ataxia Behaviour Dysphagia Pruritus Weight loss

n (%) av n (%) av n (%) av n (%) av n (%) av

VVC Any (*n*= 14) 13 (93) 2.9 6 (43) 1.2 2 (14) 1.0 8 (57) 1.4 3 (21) 2.3

AAS Any (*n*= 24) 21 (88) 2.7 16 (67) 1.2 12 (50) 1.4 10 (42) 1.7 6 (25) 2.0

TOTAL (*n* = 38) 34 (89) 2.8 22 (58) 1.2 14 (37) 1.4 18 (47) 1.6 9 (24) 2.1

Any VV (*n*= 10) 9 (90) 2.8 6 (60) 1.5 0 (0) n/a 8 (80) 1.8 0 (0) n/a

Any VA (*n*= 10) 9 (90) 2.4 5 (50) 1.0 4 (40) 1.5 4 (40) 1.8 4 (40) 1.8

Any AA (*n*= 18) 16 (89) 2.9 11 (61) 1.1 10 (56) 1.3 6 (33) 1.2 5 (28) 2.4

n, number of sheep showing each type of sign; (%) percentage; av, average score for each sign amongst sheep presenting them; n/a, not applicable as none of the sheep showed the corresponding sign.
