# Supplementary material for: Susceptibility to scrapie and disease phenotype in sheep: cross-PRNP genotype experimental transmissions with natural sources
Source: Vet Res. 2012 Jul 2;43(1):55. doi: 10.1186/1297-9716-43-55 (PMC3460791; doi:10.1186/1297-9716-43-55)
Supplement: Additional file 2 — Individual details of 38 recipients that reached clinical end point grouped by their transmission group. Individual sheep data on source of inoculum, route of inoculation, Prnp genotype, breed, survival time, clinical signs, IHC profiles and WB results [file 1297-9716-43-55-S2.doc]

**Additional File 2 Individual details of 38 recipients that reached clinical end point grouped by their transmission group.**

Inoculum Recipient ID Route Add. Breed Survival PrP^d^ Brain PrP^d^ profile Profile WB P4 Clinical signs

source 136 No. polymorph. time CNS ITNR ITGL GLAS NRPL OTHR type MW Di Mo Un ATX BEH DYS PRU WLS

**AAS** AA AAS1 po Cheviot 743 4.59 11.0 11.9 45.2 29.8 2.1 **A** 19.3 46 33 21 3 1 1 1

AAS2 po LF “ 1363 4.15 8.4 8.2 49.5 32.7 1.2 **“** 20.0 45 30 25 3 1 1

AAS3 sc “ 628 4.03 16.7 10.4 46.0 26.6 0.3 **“** 19.7 47 33 20 3 2

AAS4 sc LF “ 1118 4.60 10.0 6.9 45.9 36.2 1.0 **“** 20.2 45 32 23 3 1 1

AAS5 sc LF “ 1203 3.94 8.4 10.4 50.1 30.8 0.3 **“** 20.6 44 33 23 3 1

AAS6 sc LF “ 1198 5.32 11.3 12.3 39.3 36.5 0.6 **“** 19.7 43 31 26 3 1 1

AAS7 sc LF “ 1125 5.10 12.2 9.0 41.5 35.3 2.0 **“** 19.8 39 35 26 3 1

AAS8 po Suffolk 738 5.26 13.5 11.0 38.9 34.8 1.8 **“** 19.5 46 31 23 2 1 2 3

AAS9 po “ 680 4.09 16.0 13.0 50.3 20.0 0.7 **“** 19.8 47 32 21 1 1 1 3

AAS10 po MT “ 1162 4.21 7.8 6.6 48.9 34.7 2.0 **“** 20.2 42 30 28 3 2

VA AAS11 po Cheviot 2106 4.26 10.8 4.0 47.6 37.6 **A** 20.0 39 33 28 1 3 1

AAS12 po “ 1828 4.89 8.8 7.6 44.1 39.5 **“** 19.8 43 34 23 1 2 3

AAS13 sc LF “ 1941 4.16 10.4 3.6 43.1 41.9 1.0 **“** 20.0 40 31 29 3 1

AAS14 sc LF “ 2155 4.28 8.6 6.8 40.4 44.2 **“** 19.8 44 33 23 2 3 1 1

AAS15 po LF “ 2336 3.46 6.9 3.5 38.4 47.2 4.0 **A’** 19.8 42 32 26 3 1 2

AAS16 sc “ 1573 3.65 11.5 6.0 34.2 48.3 **“** 19.5 41 34 25 3

AAS17 sc LF “ 1638 3.10 10.3 2.6 35.5 51.6 **“** 19.3 44 33 23 3 1

VV AAS18 sc Cheviot 1650 2.86 8.7 3.5 47.9 39.2 0.7* **A** 20.2 44 33 23 1 1 3

AAS19 sc “ 1286 3.18 11.6 4.1 39.6 44.7 **A’** 19.8 38 35 27 3 3

AAS20 po “ 1371 5.63 20.8 29.3 22.0 27.9 **M’** 20.2 37 34 29 3 2

AAS21 po “ 1290 5.88 22.8 33.3 17.4 24.5 2.0 **“** 20.2 41 32 27 3 1 1

AAS22 sc “ 1385 6.10 21.2 28.7 21.6 25.2 3.3 **“** 20.6 41 31 28 3 1 1

AAS23 sc “ 1684 6.22 17.0 18.6 28.9 33.1 2.4** **U** 20.0 39 33 28 3 2 2

AAS24 sc “ 1132 4.66 24.9 9.0 32.6 32.8 0.7 **U’** 20.5 45 30 25 1 3

**VVC** VV VVC1 po Cheviot 263 5.80 28.2 38.7 9.6 23.5 **M** 21.2 39 32 29 3 1

VVC2 po “ 266 5.94 29.8 36.3 15.2 18.7 **“** 20.7 42 31 27 3

VVC3 po “ 207 5.24 30.9 39.7 10.1 19.3 **“** 21.1 42 31 27 3

VA VVC4 po LF Cheviot 371 5.24 32.6 42.6 6.3 18.5 **M** 20.1 37 31 32 3

VVC5 po “ 427 5.29 30.8 42.5 8.2 18.5 **“** 19.7 37 32 31 3 1 1

VVC6 po “ 399 4.87 29.2 43.1 9.6 18.1 **“** 19.7 37 31 32 2 1 3 2

AA VVC7 sc Suffolk 1473 5.05 19.0 14.6 38.2 27.2 1.0* **A** 19.8 46 31 23 3 2 2

VVC8 sc “ 1250 6.02 12.3 11.5 39.4 36.6 0.2 **“** 20.0 43 34 23 3 1 1

VVC9 sc MT “ 2237 3.58 9.2 5.6 47.2 38.0 **“** 20.7 41 31 28 3 1 1 1

VVC10 po “ 1451 6.53 30.7 25.6 21.0 22.2 0.5 **M’** 19.9 42 31 27 3 1 1

VVC11 po “ 1787 5.20 35.8 22.3 15.6 26.3 **“** 21.0 37 34 29 1 3

VVC12 sc “ 1297 6.47 28.9 19.3 23.0 27.1 1.7 **“** 20.2 46 30 24 3

VVC13 po “ 2003 5.10 15.1 17.8 12.8 33.6 20.7** **P** 20.2 46 31 23 3

VVC14 po LF Cheviot 1809 4.64 53.3 35.2 6.0 5.5 **CH** NR NR NR NR 3 2

ID Nos. correspond to those in main text. Add. polymorh., additional polymorphisms at codons 112 (MT) or 141 (LF) are indicated. PrP^d^ CNS correspond to the average magnitude of PrP^d^ as stated in the methods. Brain PrP^d^ profile in percentage of the different PrP^d^ types: ITNR, intraneuronal; ITGL, intraglial; GLAS, glia-associated extracellular; NRPL, extracellular in grey matter neuropil; OTHR, other types (ependymal deposits except *, vascular plaques and **, non-vascular plaques). Profiles types as described in main text. MW, molecular weight of unglycosylated band on Western blot. Di, Mo and Un, proportion of di-, mono- and unglycosylated band on WB with P4 antibody. NR, no reaction. ATX, ataxia; BEH, behaviour changes; DYS, dysphagia; PRU, pruritus; WLS, weight loss (for specific meaning of the scores refer to Table S.1.1.). Blank cells indicate absence of additional polymorphisms, PrP^d^ type or clinical sign.
